# Circulating Metabolites Associated with Body Fat and Lean Mass in Adults with Overweight/Obesity

**DOI:** 10.3390/metabo11050317

**Published:** 2021-05-13

**Authors:** Christopher Papandreou, Jesús García-Gavilán, Lucía Camacho-Barcia, Thea T. Hansen, Anders Sjödin, Joanne A. Harrold, Jason C. G. Halford, Mònica Bulló

**Affiliations:** 1 Department of Biochemistry and Biotechnology, Faculty of Medicine and Health Sciences, University Rovira i Virgili (URV), 43201 Reus, Spain; papchris10@gmail.com (C.P.); jesusfrancisco.garcia@iispv.cat (J.G.-G.); 2 Institute of Health Pere Virgili—IISPV, University Hospital Sant Joan, 43201 Reus, Spain; 3 Consorcio CIBER, M.P. Fisiopatología de la Obesidad y Nutrición (CIBEROBN), Instituto de Salud Carlos III, 28029 Madrid, Spain; lucamacho@gmail.com; 4 Department of Psychiatry, University Hospital of Bellvitge-IDIBELL, Hospitalet de Llobregat, 08908 Barcelona, Spain; 5 Section for Obesity Research, Department of Nutrition, Exercise and Sports, University of Copenhagen, 2200 Copenhagen, Denmark; tha@nexs.ku.dk (T.T.H.); amsj@nexs.ku.dk (A.S.); 6 Department of Psychology, Institute of Population Health, University of Liverpool, Liverpool L69 3GL, UK; harrold@liverpool.ac.uk (J.A.H.); j.halford@leeds.ac.uk (J.C.G.H.); 7 Appetite Control & Energy Balance Research Group, School of Psychology, Faculty of Medicine & Health, University of Leeds, Leeds LS2 9JT, UK

**Keywords:** metabolomics, body composition, fat mass, lean mass, SATIN

## Abstract

The interplay between fat mass and lean mass within human metabolism is not completely understood. We aimed to identify specific circulating metabolomic profiles associated with these body composition compartments. Cross-sectional analyses were conducted over 236 adults with overweight/obesity from the Satiety Innovation (SATIN) study. Body composition was assessed by dual-energy X-ray absorptiometry. A targeted multiplatform metabolite profiling approach was applied. Associations between 168 circulating metabolites and the body composition measures were assessed using elastic net regression analyses. The accuracy of the multimetabolite weighted models was evaluated using a 10-fold cross-validation approach and the Pearson’s correlation coefficients between metabolomic profiles and body compartments were estimated. Two different profiles including 86 and 65 metabolites were selected for % body fat and lean mass. These metabolites mainly consisted of lipids (sphingomyelins, phosphatidylcholines, lysophosphatidylcholines), acylcarnitines, and amino acids. Several metabolites overlapped between these body composition measures but none of them towards the same direction. The Pearson correlation coefficients between the metabolomic profiles and % body fat or lean mass were 0.80 and 0.79, respectively. Our findings suggest alterations in lipid metabolism, fatty acid oxidation, and protein degradation with increased adiposity and decreased lean body mass. These findings could help us to better understand the interplay between body composition compartments with human metabolic processes.

## 1. Introduction

Increased prevalence of obesity, assessed by body mass index (BMI), is one of the largest health concerns globally being a major risk factor for a number of prevalent chronic diseases [1]. However, BMI is an indirect estimate of adiposity as it does not distinguish between fat mass and lean mass. An abundance of evidence indicates an increased risk of cardiometabolic diseases [2,3] and mortality [4] for those who have an increased body fat and/or reduced lean mass. However, the underlying mechanisms linking these two compartments of body composition with health outcomes are not fully understood. It has been suggested that several factors including genetic, physiologic, metabolic, and behavioral may explain this link [5]. Prior studies have identified different circulating metabolites such as amino acids, acylcarnitines, or lipid species associated with body fat [6,7,8], lean mass [7,9,10,11], and metabolic risk [6,12,13]. However, to date, limited metabolomic-analysis has been conducted using combinations of different metabolomic platforms to cover a wide range of metabolites and examine their association with these body composition compartments. A comprehensive metabolite profiling (metabolomics) may provide a deeper understanding of the interplay between fat mass and lean mass with human metabolism. The exclusion of participants with manifestation ofeither cardiometabolic diseases also reduces unwanted confounding when investigating body composition measures in relation to metabolomic profiles [14].

Therefore, we used a multiplatform metabolomics approach to identify circulating metabolomics profiles associated with body fat and lean mass in participants with overweight/obesity included in the EU project Satiety Innovation (SATIN) study.

## 2. Results

The general characteristics of the 236 participants are summarized in Table 1. The mean age was 46.4 years, with a mean body mass index (BMI) of 31.1kg/m^2^. The majority of women were pre-menopausal (>90%). The mean % body fat was 42.0, and the lean mass was 47.2 kg. Pearson’s correlation analysis revealed that % body fat was significantly correlated with lean mass (r = −0.71, *p*-value: <0.001).

### 2.1. Circulating Metabolites Associated with Body Composition Measures

**Figure 1:**
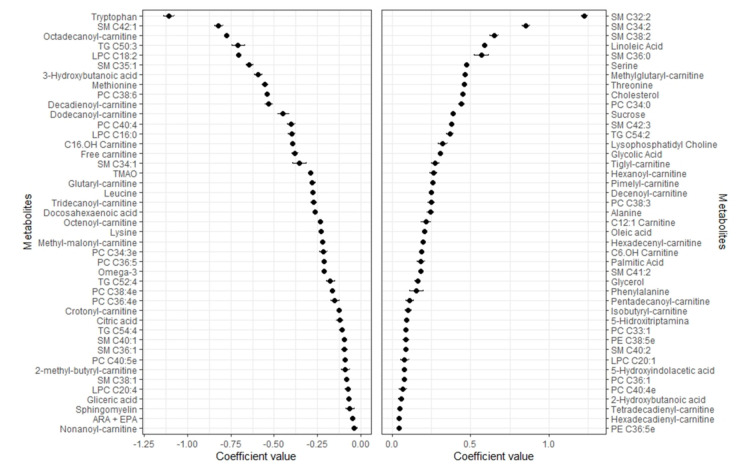
Coefficients (mean ± SD) for the metabolites selected 9–10 times in the 10-fold CV linear elastic regression and associated with % body fat.

**Figure 2:**
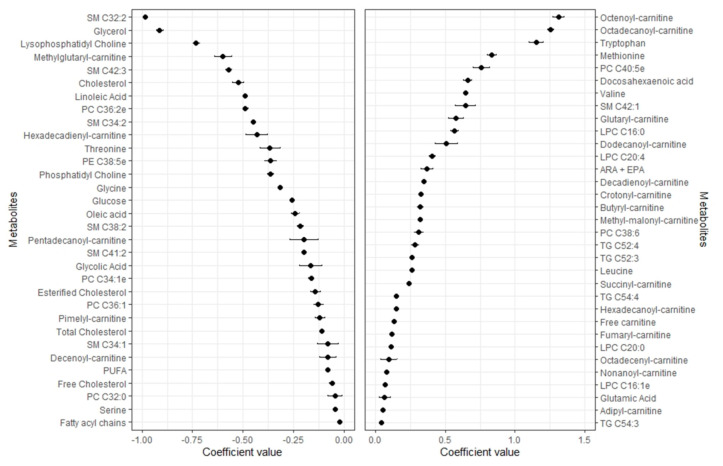
Coefficients (mean ± SD) for the metabolites selected 9–10 times in the 10-fold CV linear elastic regression and associated with lean mass.

Of the 168 metabolites used in the analyses, the elastic net regression model selected 86 and 65 metabolites for % body fat and lean mass, respectively (Figure 1 and Figure 2). The selected metabolites shown in the respective Figure 1 and Figure 2 were ranked from the highest to the lowest elastic net positive and negative regression coefficients.

Mean and SD of the set of 86 metabolites selected 9–10 times in the 10-fold CV elastic linear regression procedure (using lambda.min). Metabolites with negative coefficients (m = 43) are plotted in the left part, whereas those with positive coefficients (m = 43) are shown in the right part. Abbreviations: ARA + EPA, Arachidonic acid + Eicosapentaenoic acid; LPC, Lysophosphatidylcholine; PC, Phosphatidylcholine; PE, Phosphatidylethanolamine; SM, Sphingomyelin; TG, Triacylglycerides; TMAO, Trimethylamine N-oxide.

Mean and SD of the set of 65 metabolites selected 9–10 times in the 10-fold CV elastic linear regression procedure (using lambda.min). Metabolites with negative coefficients (m = 32) are plotted in the left part, whereas those with positive coefficients (m = 33) are shown in the right part. Abbreviations: ARA + EPA, Arachidonic acid + Eicosapentaenoic acid; LPC, Lysophosphatidylcholine; PC, Phosphatidylcholine; PE, Phosphatidylethanolamine; PUFA, polyunsaturated fatty acids; SM, Sphingomyelin; TG, Triacylglycerides.

### 2.2. Metabolomic Profile of Body Fat

Forty-three metabolites were positively associated with % body fat, and 43 were negatively associated. High positive regression coefficients were found for sphingomyelins (SMs: C32:2, C34:2, C38:2, C34:0), linoleic acid, serine, threonine, alanine, six carnitines (methylglutaryl-, tiglyl-, hexanoyl-, pimelyl-, decenoyl-, hexadecenyl-), phosphatidylcholine (PC: C38:3), total lysophosphatidylcholine (LPC), TG C54:2, sucrose, glycolic acid, followed by other carnitines, several PCs, SMs, and phosphatidylethanolamines (PEs), oleic acid, palmitic acid, glycerol and phenylalanine.The highest negative regression coefficient was found for tryptophan followed by SM C42:1, octadecanoyl-carnitine, TG C50:3, LPC C18:2, SM C35:1, 3-hydroxybutanoic acid, methionine, PCs (C38:6, C40:4), eight other carnitines (decadienoyl-, dodecanoyl-, C16 OH, free, glutaryl-, tridecanoyl-, octenoyl-, methyl-malonyl-), LPC C16:0, SM C34:1, TMAO, leucine, other carnitines, several PCs and SMs, omega-3 fatty acids, and citric acid. Other LPC species negatively associated with body fat were C16:0 and C20:4.

### 2.3. Metabolomic Profile of Lean Mass

Out of the 65 metabolites associated with lean mass, 33 had positive and 32 negative regression coefficients. The highest positive regression coefficients were observed for two carnitines (octenoyl-, octadecanoyl-) and tryptophan followed by methionine, PC C40:5e, docosahexaenoic acid, valine, SM C42:1, glutaryl- and dodecanoyl-carnitine, LPCs (C16:0, C20:4, C20:0, C16:1e), several other carnitine species, leucine, and glutamic acid. High negative regression coefficients were obtained for SMs C32:2, glycerol, lysophosphatidylcholinemethylglutaryl-carnitine, SM C42:3 cholesterol, linoleic acid, several PCs, SMs, glycine, glucose, oleic acid, and other carnitines.

### 2.4. Pearson Correlations between Metabolomic Profiles and Body Compartments

In the training set, the unbiased metabolomic profiles acquired using the 10-fold cross-validation approach was strongly correlated with % body fat (r = 0.80, *p*-value: <0.001) and moderately with lean mass (r = 0.78, *p*-value: <0.001) (Table 2).

Sensitivity analysis adjusting for age and sex showed that 16 metabolites of the 86 previously selected from the unadjusted model were associated with % body fat (Supplemental Figure S1), while 9 metabolites were selected for lean mass (Supplemental Figure S2).

## 3. Discussion

Using baseline data from the SATIN study and performing a comprehensive metabolite profiling, we identified two different metabolomic profiles associated either with % body fat or with lean mass. These metabolites mainly included lipid species and acylcarnitines suggesting lean tissue- and adipose-related alterations in lipid metabolism with increased adiposity and decreased lean mass. Furthermore, some metabolites associated with measures of body fat were consistently associated with lean mass. This may reflect correlations between these body composition measures. Interestingly, the identified multimetabolite models exhibited strong correlations with the body composition compartments.

A previous study that performed a lipidomic analysis in plasma of adults with obesity or normal weight revealed LPC as the most significant lipid associated with obesity [15]. In our study, most of the associations between these lipid species were observed for body fat. Noticeably, among lipids, the most prominent associations were for SM C32:2 with both body compartments but in opposite directions. This SM is not unknown in obesity research, as it has been shown to be associated with BMI in young Australian adults [16] and in Mexican American adults [17]. We also observed, for the first time, that the SM C32:2 was accompanied by other SMs with two double bonds (i.e., SM C34:2, SM C38:2, SM C41:2) and positively associated with % body fat, while negatively with lean mass. On the other hand, SMs with one double bond (i.e., SM C42:1, SM C35:1, SM C34:1, SM C40:1, SM C38:1) were negatively associated with body fat, whereas SM C42:1 was positively associated with lean mass and these associations have not previously reported. Previous experimental studies suggest that sphingolipids may play a role in adipogenesis by directing the adipocyte toward storage [18]. Given the role of circulating sphingolipids in atherosclerosis development [19], the increased circulating concentrations of SMs with two double bonds and decreased concentrations with one double bond associated with increased adiposity we found in our analysis could partially explain the increased cardiovascular risk associated with excessive adiposity. However, the exact molecular species could not be specified—a known pitfall of most screening methods. PCs, the most abundant phospholipids in mammalian membranes and direct substrates for the formation of SMs, were mostly associated with body fat. Our results in relation to LPC species and lower % body fat or higher lean mass are in line with previous findings from the comparison between lean and non-diabetic individuals with obesity [15,20].

A metabolite profile, including 24 and 20 acylcarnitines, was related to% body fat and lean mass, respectively. Most of these compounds were consistently associated with both body composition measures but in the opposite direction. Our results confirm previously positive associations between twoacylcarnitines (hexanoylcarnitine and hexadecenoylcarnitine) and % body fat [8]. However, our associations of octenoylcarnitine and tetradecadienylcarnitine with % body fat were not in the same directions as reported by Mai and colleagues [8]. It is likely that the higher body fat correlates with an upregulated beta oxidation of fatty acids, which predominantly leadsto higher amounts of short- or medium- chain-acylcarnitines.

Among the fatty acids assessed, docosahexaenoic acid was positively associated with lean mass and negatively with body fat. A previous study in children with obesity showed inverse associations between docosahexaenoic acid in red blood cells and % body fat [21]. On the contrary, the omega-6 fatty acid, linoleic acid, which has been identified as obesogenic [22], was associated with increased body fat and decreased lean mass. Beyond similarities with previous studies, we also found oleic acid to be associated with both body composition measures in similar directions as linoleic acid. Oleic acid has been shown to stimulate adipogenesis in hen preadipocytes by increasing the expression of key adipogenic transcription factors such as CCAAT/enhancer binding protein, alpha, or fatty acid binding protein 4 [23].

Besides the altered fatty acid oxidation with increased adiposity, changes in amino acid metabolism have also been reported. In a small cross-sectional study of Japanese adults, higher levels of branched-chain amino acids, lysine, tryptophan, cystine, and glutamate, while lower levels of asparagine, citrulline, glutamine, glycine, and serine were associated with obesity [24]. In a larger study, higher levels of several amino acids were found in obese versus lean Japanese subjects [25]. Similar to our study, Murphy and colleagues reported associations of several amino acids (tryptophan, methionine, valine, leucine, glutamic acid) with lean mass [7]. Amino acids have well-established roles in maintenance of muscle nitrogen balance [26]. On the other hand, serine, threonine, alanine, and phenylalanine were associated with increased body fat. It is possible that the greater the adiposity the higher the protein degradation increasing the circulating concentrations of these amino acids [27].

Our study has several strengths. A comprehensive metabolite profiling was performed using combinations of different metabolomic platforms to quantitatively analyze a wide range of metabolites. The body composition was assessed by dual-energy X-ray absorptiometry (DXA), an objective, gold-standard method for measuring adiposity. Our study participants were overweight/obese but free of chronic diseases and were non-smokers, all factors that may affect the concentrations of these metabolites. Concerning limitations, we evaluated a sample of individuals mainly consisting of women with overweight/obesity and without comorbidities that could limit the generalizability of our results to other populations. However, the replication of prior associations with % body fat and lean mass [25] suggests that some of the findings may be not specific to our population characteristics. Second, due to the cross-sectional design, causation and direction of causality cannot be inferred, therefore both directions are currently plausible and require further investigation. Third, the relatively small sample size did not allow us to conduct stratified analyses by age and sex and thus examine whether the obtained metabolic profile differ depending on ageor sex of the participants.

## 4. Materials and Methods

### 4.1. Study Design and Participants

The present study was nested within the SATIN work package 5, including 236 participants from Denmark and Spain. Detailed information about study design, visits, and methods has been previously published [28,29]. The SATIN study was designed as a two-phase, double blinded parallel, randomized multicenter trial. Eligible participants were men and women (20–65 years) with a BMI of 27.0 to 35.0 kg/m^2^, fat mass ≥23%, and without comorbidities at baseline. Participants with significant weight changes (±3 kg in the last three months), severe chronic medical conditions (type 1 or 2 diabetes, cardiovascular diseases, hypertension, chronic kidney diseases, liver diseases, active inflammatory bowel diseases, cancer, bariatric surgery and other interventions, psychological or behavioral problems, psychiatric disorders), drug addictions, regular alcohol consumption above recommendations and current smoking (including smoking cessation within the last three months prior to study) were excluded from the study. After an initial 8-week low-calorie diet (Modifast^®^, Nutrition et Santé, France), participants who reached at least an 8% weight reduction, after a 7–10 days run-in period for diet stabilization, were randomly allocated in a 1:1 ratio to the second part of the study (weight-loss maintenance period) following one of the two intervention: (1) Regular diet including an active satiety-enhancing product (active intervention group) or (2) regular diet including a similar control product without satiety enhancing properties (control group) for 12 weeks. In the current analysis, participants with available blood samples and DXA data at the beginning of the first period (before weight loss diet) were considered.

The study was conducted in accordance with the ethical principles set forth in the current version of the Declaration of Helsinki (Fortaleza, Brazil, October 2013). The protocol was approved by the local institutional review boards and Ethics Committees of all the recruiting centres (the Municipal Ethical Committee of Copenhagen/Scientific Ethics Committee of the Metropolitan regions of Denmark (journal no. H-15008553), the Danish Data Protection Agency (journal no. 2015-57-0117), and the Ethical Committee for Clinical Research (journal no. 15-07-30/7assN2) and all participants provided written informed consent. This trial was registered in: clinicaltrials.gov (accessed on 12 May 2021) (identifier: NCT02485743). In addition, all study procedures were aligned between sites before initiation of the study and on-site monitoring visits were carried out by an independent monitor.

### 4.2. Anthropometry and Routine Biochemical Measurements

All anthropometric measures were performed by trained staff. Height without shoes was measured to the nearest 0.5 cm and body weight while wearing light clothing and having emptied the bladder was measured to the nearest 0.1 kg. Both were measured in metric units and using a wall-mounted stadiometer (Seca, Hamburg, Germany) and digital calibrated scales (Copenhagen: Lindell Tronic 8000, SamhallLavi; Reus: Tanita SC-331S, Tanita Corporation of America Inc., Arlington Heights, IL, USA), respectively, and used to determine BMI.

Blood samples were collected in fasting conditions before the initial weight-loss period. Plasma was obtained, aliquot, and stored at −80 °C until the metabolomics analysis. A general routine biochemical analysis including glucose and lipid profile wasperformed using standard enzymatic automated methods (COBAS; Roche Diagnostics Ltd., Rotkreuz, Switzerland).

### 4.3. Body Composition Assessment

A Lunar Prodigy X-ray Bone Densitometer (Lunar Prodigy Primo, GEHealthcare, Little Chalfont, UK, in participants from Reus and GE Lunar iDXA, Encore software version 16.2 in participants from Copenhagen) was used to acquire DXA scans and assess body fat, and lean mass. The DXA scan was performed in fasting conditions with the participants only wearing light clothing and after emptying the bladder. The DXA scan was performed according to the manufacturer’s instructions for the device and calibrated according to manufacturer’s instructions. The same device and software were used for the same participant throughout the entire study. In women, and according to local requirements and procedures, a pregnancy test (by urine stick) was performed before each scan, or the women were asked to clearly state lack of pregnancy in Denmark. In case of a positive test/statement the scan was not conducted. Body fat is expressed relative to total body mass as percentage, and lean mass as kg as is standard practice [30,31].

### 4.4. Multiplatform Targeted Metabolomics

Metabolites were analyzed using a multiplatform approach previously published [32]. These platforms comprise proton nuclear magnetic resonance (^1^H-NMR), liquid chromatography coupled to high resolution mass spectrometry (LC-HRMS), and gas chromatography coupled to high-resolution mass spectrometry (GC-HRMS). Fasting blood samples for GC-HRMS analysis were dried and stored at −80 °C until analysis.

### 4.5. Automated Plasma Sample Extraction

For metabolite extraction, the Bravo Automated Liquid Handling Platform from Agilent Technologies was used to extract plasma samples in 96-well format plates.

For GC-HRMS analysis, a protein precipitation extraction will be made by adding 400 of μL MeOH: H_2_O (8:1) mixture to a volume of 100 μL of plasma. The mixture was stirred and centrifuged and the supernatants were collected in new 96-well plates that contain internal standard mixture. This plate was evaporated to dryness with a vacuum centrifugation system (Speed Vac) and dried extracts were reconstituted with 30 μL of methoxyamine and incubated during 90 min at 37 °C. Finally, the metabolites were sylilated with 45 μL of MSTFA + 1% TMCS at room temperature during 60 min.

For LC-HRMS analysis and NMR analyses, lipidic fraction was obtained by a liquid-liquid extraction using a methanol/methyl-tert-butyl ether mixture. These solvents were automatically and sequentially added to a volume of 100 μL of plasma with agitation stages between them and final centrifugation to promote phase separation. Then, a small aliquot of the supernatant (organic phase) was dispensed and diluted 1:10 with methanol in a new 96-well plates containing deuterated internal standards for each family of lipids (Lipidomix SPLASH from Avanti Polar Lipid) for lipidomic analysis using liquid chromatography coupled to a time of flight high resolution mass spectrometry (LC-HRMS).

For lipidomic analysis by NMR, a second aliquot of the supernatant (organic phase) was dispensed in new 96 well plates that was evaporated to dryness with Speed Vac. Afterwards, they were reconstituted with a solution of CD_3_Cl: CD_3_OD with 4% D_2_O and 0.01% TMS (0.067 mM, Eretic Signal 6.166 mM) and analyzed by proton NMR (^1^H-NMR) [33].

#### 4.5.1. ^1^H-NMR

Samples were prepared following the procedure previously published [29]. NMR spectra were recorded at 300 K on an Avance III 600 spectrometer (Bruker, Germany) at 600.20 MHz using a 5 mm PBBO gradient probe. Lipid samples were measured and recorded in PROCNO 11 using a simple pre-saturation sequence (recycle delay (RD)–90°–ACQ pre-saturation pulse (zgpr) program). Specific ^1^H regions of diacylgycerols, triglycerides, and total lipids based on terminal methyl and methylene signals were identified in the spectra using a comparison in the AMIX 3.9 software (Bruker, Germany) after pre-processing and visual checking of the NMR dataset.

#### 4.5.2. LC-HRMS

The lipid species in plasma samples were determined by ultra-high performance liquid chromatography (UHPLC) coupled to quadrupole-time of flight (qTOF) high resolution mass spectrometry (MS) (6550 iFunnel series, Agilent Technologies, Spain) (following the procedure described in Hernandez-Alonso et al. [34]). Lipids were separated in a C18 reversed phase column (Kinetex C18-EVO from Phenomenex) and a ternary mobile phase (water/methanol/2-propanol) was used. The lipids measurements were generated from specific RT, isotope peaks relation and the most intense adduct form observed. Each lipid was quantified with an internal standard calibration method using one analytical standard and one deuterated internal standard for each lipid family (lysophosphatidylcholines, phosphatidylcholines, sphingomyelins, and triglycerides). Specific vendor software was used (Quantitative Mass Hunter from Agilent).

#### 4.5.3. GC-HRMS

Following the procedure described in Hernandez-Alonso et al. [34], samples were analyzed in a 7890A Series GC coupled to a triple quadrupole (QqQ) (7000 series; Agilent Technologies, Barcelona, Spain) using the J&W Scientific HP5-MS (30 m × 0.25 mm i.d., 0.25 µm film; Agilent Technologies, Barcelona, Spain) chromatographic column and helium as a carrier gas. Ionization was carried out with electronic impact recording data in “Full Scan” mode.

Metabolite measurements were based on specific RT plus an ion fragmentation pattern. Quantification was performed by internal standard calibration, using the corresponding analytical standard for each determined metabolite (succinic d4 acid, glycerol 13C3, norvaline, L-methionine-(carboxy-13C, methyl-d3), D-glucose 13C6, myristic-d27 acid, and alpha-tocopherol d6), and a deuterated internal standard depending on the family of metabolite.

### 4.6. Statistical Analyses

Characteristics of study participants were described as means (SD) for quantitative traits and percentages for categorical variables. Individual metabolites with equal or more than 20% missing values were excluded, otherwise data were imputed using the random forest imputation method “missForest” function from the “randomForest” v 4.6-14 R package. Subsequently, 9 metabolites were excluded from the 178 quantitative panel metabolites included in the study, leaving 168 metabolites for further analyses. Rank-based inverse normal transformation was applied to the metabolomics data to improve normality. Gaussian regression with an elastic net penalty was used to build a multimetabolite model for each of the body composition measures (“caret” v 6.0-84 and “glmnet” v 3.0-2 R package). We performed 10-fold cross-validation (CV) to find the optimal value of the tuning parameter that result in a mean squared error within 1-SD of the minimum [35]. The performance of the model was examined based on parameters of “lambda.min”. The multimetabolite model was computed as the weighted sum of the selected metabolites with weights equal to regression coefficients from the model.

A 10-fold cross-validation (CV) approach was performed splitting the whole dataset into training and validation sets (80% and 20%, respectively). Subsequently, in the training set, we applied this approach to obtain the performance of the model without overfitting. Models were optimized using argument best Tune of the “caret” R package. In order to report the coefficients from each CV iteration, s = “lambda.min” was selected as it gives the minimum mean CV error. The alpha parameter was also estimated using 0.1 increments from 0 (i.e., Ridge regression) to 1 (i.e., Lasso regression). The alpha value of the model with the best predicting accuracy in the validation sets was 0.8 and the lambda.min values were 1.119 and 0.568 for fat mass and lean mass models, respectively. Weighted models for each training-validation datasets (i.e., for every 80–20 split datasets) were constructed using the metabolite coefficients obtained from the elastic net regression of each training set. Pearson correlations were calculated to evaluate the performance of the multimetabolite model in assessing % body fat or lean mass in the validation set. For reproducibility purposes, we presented the regression coefficients using 10 iterations of the 10-CV elastic regression approaches in the whole dataset. To address potential confounding effects of age and sex on the association between metabolites and body compartments, we conducted a sensitivity analysis by adding them as covariates. All the analyses were performed using R statistical software (v 3.6.1).

## 5. Conclusions

In conclusion, this study identified specific profiles of 86 metabolites associated with % body fat and 65 metabolites associated with lean mass in a sample of adults with overweight/obesity. These findings suggest alterations in lipid metabolism, fatty acid oxidation, and protein degradation with increased adiposity and decreased lean mass and contribute to further our understanding of the interplay between body compartments and metabolic status that could link body composition with metabolic disorders.

## Figures and Tables

**Table 1:** Characteristics of study participants.

Characteristics	(*n* = 236)
Age, years	46.4 ± 10.7
Women sex, N (%)	184 (78)
Weight, kg	87.5 ± 11.2
BMI, kg/m^2^	31.1 ± 2.2
Body fat, %	42.0 ± 5.6
Lean mass, kg	47.2 ± 9.2
Glucose, mg/dL	93.3 ± 11.0
Total cholesterol, mg/dL	196.0 ± 34.9
HDL-C, mg/dL	55.7 ± 15.3
LDL-C, mg/dL	119.9 ± 30.5
Triglycerides, mg/dL	102.3 ± 48.9

Data shows mean ± SD or number (%); Abbreviations: BMI, body mass index; HDL-C, high-density lipoprotein cholesterol; LDL-C, low-density lipoprotein.

**Table 2:** Ten-fold CV Pearson (95% CI) correlations between the multimetabolite model and % body fat and lean bodymass.

	% Body Fat	*p*-Value	Lean Mass	*p*-Value
Pearson’s correlation coefficient (95%CI)	0.80 (0.75, 0.84)	<0.001	0.78 (0.72, 0.83)	<0.001

All metabolites were obtained 10 times in the cross-validation procedure for the elastic net Gaussian regression using “lambda.min” option. Abbreviations: CV, cross-validated.

## Data Availability

Data generated and analysed in the framework of the SATIN Consortium are not publicly available due to national data regulations and for ethical reasons, because study participants only gave their consent for the use of their data by the SATIN Consortium investigators. However, collaboration for data analyses can be requested by sending a letter to the corresponding author. The request would be evaluated by the SATIN Steering Committee for deliberation.

## Supplementary Materials

The following are available online at https://www.mdpi.com/article/10.3390/metabo11050317/s1, Figure S1: Coefficients (mean ± SD) for the metabolites selected 9–10 times in the 10-fold CV linear elastic regression and associated with% body fat adjusted for age and sex, Figure S2: Coefficients (mean ± SD) for the metabolites selected 9–10 times in the 10-fold CV linear elastic regression and associated with lean mass adjusted for age and sex.
