# Supplementary material for: Circulating Metabolites Associated with Body Fat and Lean Mass in Adults with Overweight/Obesity
Source: Metabolites. 2021 May 13;11(5):317. doi: 10.3390/metabo11050317 (PMC8153621; doi:10.3390/metabo11050317)
Supplement: Supplementary file 1 [file metabolites-11-00317-s001.zip › metabolites-1206784-supplementary.pdf]

Supplemental Figure 1. Coefficients (mean ± SD) for the metabolites selected 9-10 times in the 10-fold CV linear elastic regression and associated with % body fat adjusted for age and sex.

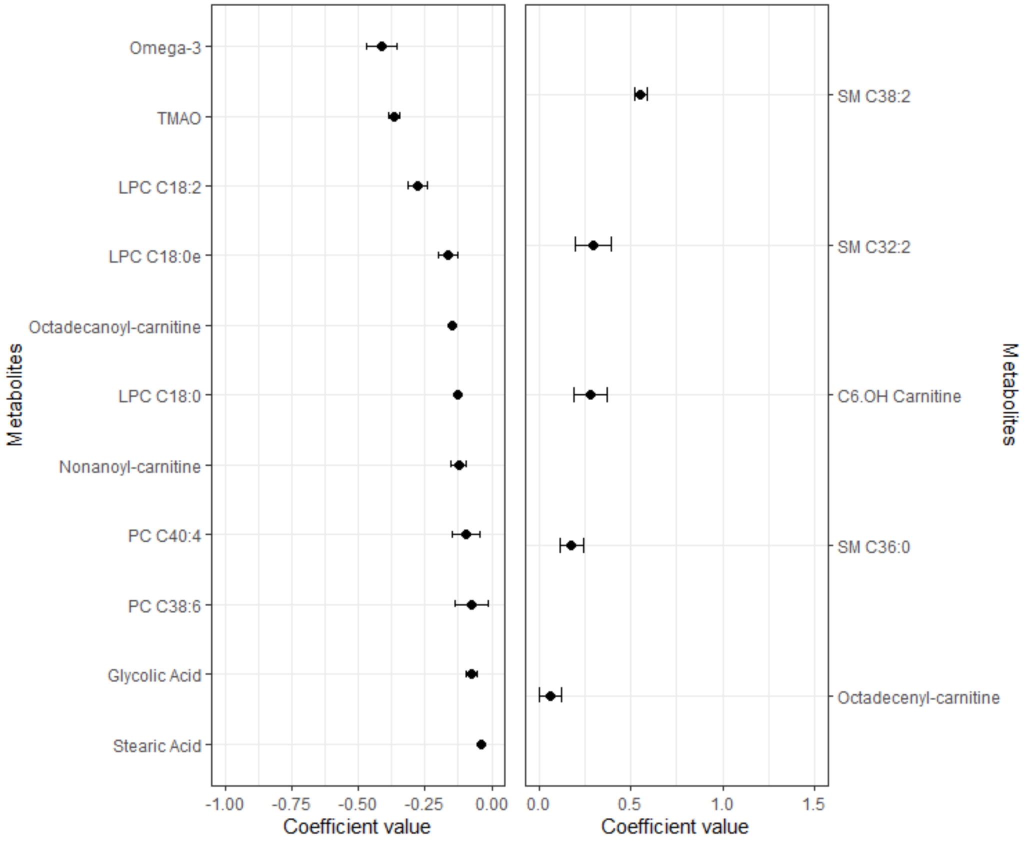

Mean and SD of the set of 16 metabolites selected 9-10 times in the 10-fold CV elastic linear regression procedure (using lambda.min). Metabolites with negative coefficients (m = 11) are plotted in the left part, whereas those with positive coefficients (m = 5) are shown in the right part. Abbreviations: LPC, Lysophosphatidylcholine; PC, Phosphatidylcholine; PE, Phosphatidylethanolamine; SM, Sphingomyelin; TMAO, Trimethylamine N-oxide.

Supplemental Figure 2. Coefficients (mean ± SD) for the metabolites selected 9-10 times in the 10-fold CV linear elastic regression and associated with lean mass adjusted for age and sex.

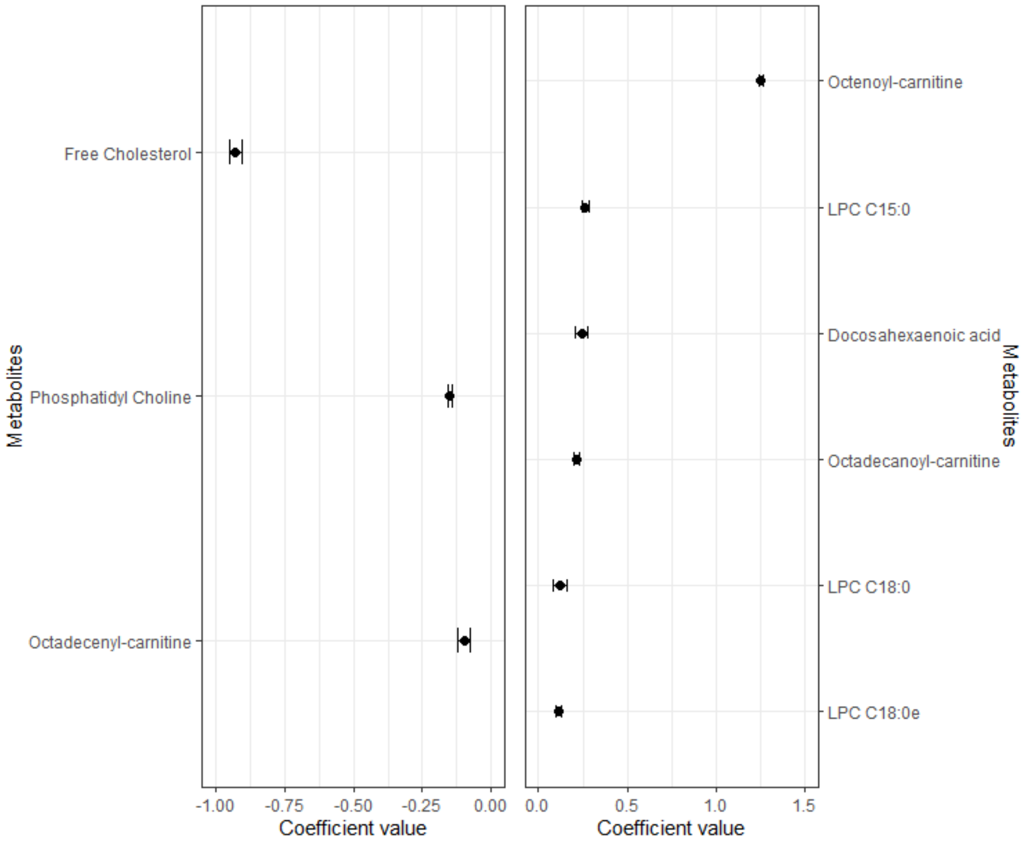

Mean and SD of the set of 9 metabolites selected 9-10 times in the 10-fold CV elastic linear regression procedure (using lambda.min). Metabolites with negative coefficients (m = 3) are plotted in the left part, whereas those with positive coefficients (m = 6) are shown in the right part. Abbreviations: PC, Phosphatidylcholine.
